# Spinal Cord Infarct Due to Fibrocartilaginous Embolism in an Adolescent Boy: A Case Report and Literature Review

**DOI:** 10.7759/cureus.37319

**Published:** 2023-04-09

**Authors:** Said A Al-Farsi, Haifa Al-Abri, Eiman Al-Ajmi, Abdullah Al-Asmi

**Affiliations:** 1 Neurology, Sultan Qaboos University, Muscat, OMN; 2 Radiology and Molecular Imaging, Sultan Qaboos University, Muscat, OMN

**Keywords:** oman, adolescent, anterior spinal artery syndrome, spinal cord infarction, fibrocartilaginous embolism

## Abstract

Fibrocartilaginous embolism (FCE) is one of the rare causes of acute spinal cord infarction. We report the case of a previously healthy 14-year-old boy with this condition. A few hours after lifting heavy objects, he developed sudden quadriparesis. On examination, he had asymmetric hypotonic quadriparesis and normal dorsal column function but absent spinothalamic function in all limbs with sensory level to shoulder. Magnetic resonance imaging (MRI) of the spine confirmed the diagnosis of spinal infarction secondary to FCE. The patient initially received methylprednisolone and plasma exchange. A follow-up visit after neurorehabilitation showed improvement but with residual neurological deficit. Although FCE is rare, it should be kept as one of the differential diagnoses of an acute neurological deficit of the spinal cord.

## Introduction

Fibrocartilaginous embolism (FCE) is a rare cause of spinal cord infarction, where dislodged material from the fibrocartilaginous pulposus nucleus causes occlusion in a spinal cord blood vessel [1]. There are different pathways in which FCE can access the bloodstream, including persistence or revascularization of intervertebral disc vasculature or the formation of Schmorl’s nodes [1]. Extremely rare in childhood, FCE cases increase in prevalence from adolescence and adulthood due to age-related changes in spinal cord vasculature [1,2]. Most FCE events present quite suddenly with variable neurological signs and symptoms, usually after minor trauma or weightlifting [2]. Due to the rarity of FCE, the similarity of its clinical courses and presentations with other neurological diseases, and the lack of definite diagnostic tools, it is often misdiagnosed and sub-optimally managed.

## Case presentation

A 14-year-old boy presented with his left side completely paralyzed and minimal movement in his right upper and lower limbs. The previous evening, while playing with his mobile phone, he started experiencing mild neck pain radiating to his shoulders. Within a few minutes, he developed a sudden progressive descending paralysis and numbness that progressed from his upper limbs to his lower limbs. He also developed urinary incontinence, poor cough effort, and episodes of choking on liquids. Within an hour, he was admitted to the nearby regional hospital, which referred him to us the next day.

The patient denied any previous history of headache, fever, flu-like symptoms, diarrhea, allergy, or trauma. He was not on any regular medication. His family history also was negative for similar symptoms, neurological diseases, or malignancy.

**Figure 1:**
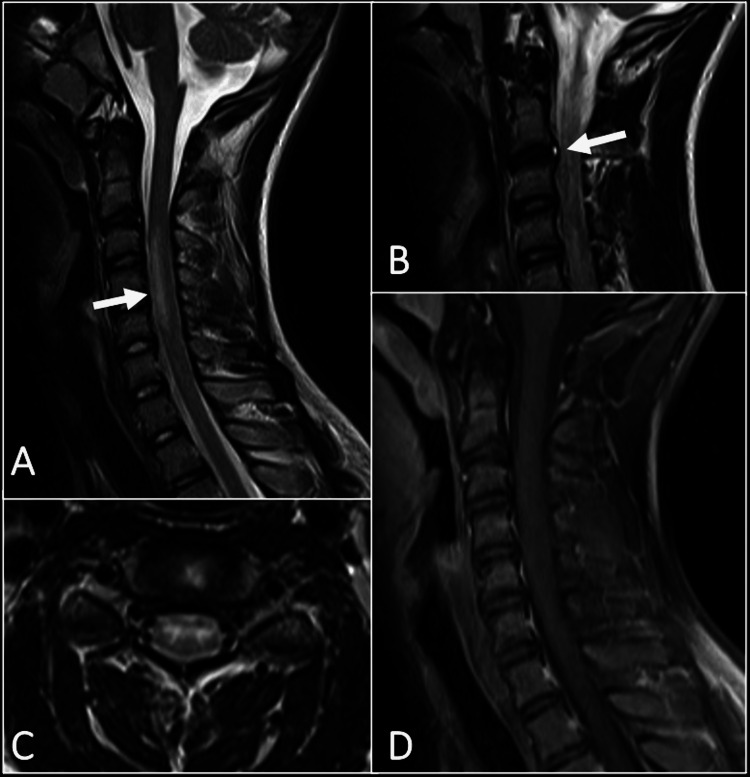
Initial MRI: (A) Sagittal T2 weighted image of the cervical spine shows intramedullary signal abnormality from C3 to C5 in the anterior cord with mild swelling of the cord (arrow). (B) There are isolated reduced signal intensities of the intervertebral disc at C2-C3 with posterior hyperintensity in keeping with the annular tear (arrow). (C) The axial T2 image of the cord at C3-C4 shows that the signal abnormality is involving the central gray matter. (D) No abnormal enhancement is seen in the cord in this post-contrast image.

The referring regional hospital had sent us the magnetic resonance image (MRI) of the patient’s spine taken within a few hours of the onset of symptoms. The MRI showed swelling and T2 hyperintensity in the cord from C3 to C5, with central gray matter involvement in the axial T2 images. There was isolated reduced T2 signal intensity in the intervertebral disc at C2-C3, with T2 hyperintensity in the disc posteriorly, suggesting an annular fissure. There was no evidence of abnormal enhancement. Other intervertebral discs were unremarkable. Diffusion-weighted images were not available (Figure 1).

On examination, the patient was a thin-built adolescent lying quietly in bed, alert and oriented, with no outward signs of distress. He was afebrile and hemodynamically stable. He did not have neck stiffness or rigidity with normal neck flexion and extension. Kernig’s and Brudzinski’s signs were negative. Cranial nerves were normal. The shoulder shrug was strong and symmetric. He was hypotonic in all limbs and bilaterally areflexic except in ankles. Power was 2/5 in the right upper limb, 3/5 in the right lower limb, 0/5 in the left upper limb, and 1/5 in the left lower limb. He could feel a vibration and sense joint position and light touch in all limbs but could not feel the temperature change except at the shoulders. His torso was numb, and the best sensation was reached at shoulder level. Abdominal reflexes were absent.

As the patient was maintaining oxygen saturation despite poor cough effort and tachypnea, he was kept in a high-dependency unit with close follow-up from the intensive care unit team.

The results of hematological, biochemical, and cerebrospinal fluid analyses were unremarkable. Workups for autoimmunity and hypercoagulable state yielded negative results. The results were also normal for computerized tomography (CT) of the brain and neck, CT angiography, echocardiography, and 24-hour ECG Holter.

Among the top treatable differential diagnoses of this patient’s condition was transverse myelitis. Accordingly, he was given an intravenous course of methylprednisolone and sessions of plasma exchange with no to minimal improvement.

Seven days post-admission, the patient recalled that he had engaged in the lifting of heavy cement blocks a few hours before his neurological symptoms began. With this new information, his MRI findings were reviewed, and a diagnosis of anterior spinal artery infarct due to a fibrocartilaginous embolism (FCE) was reached. The ongoing treatment (methylprednisolone and plasma exchange) was stopped; the patient was started on prophylactic aspirin and advised on long-term rehabilitation.

**Figure 2:**
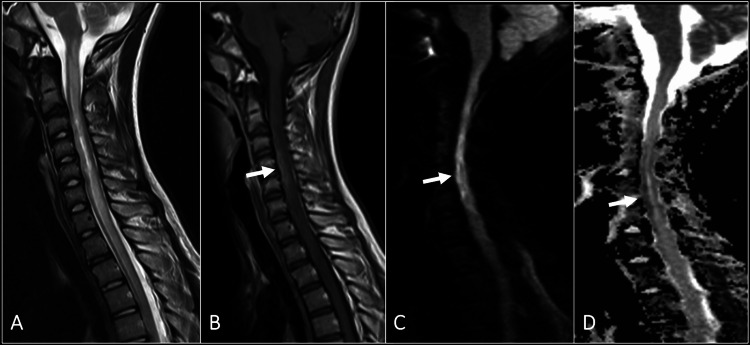
The follow-up MRI after two weeks from the onset: (A) Sagittal T2 image shows interval worsening of the signal abnormality and the swelling of the spinal cord. (B) There is an abnormal enhancement in the anterior cord, which is localized at the same area of abnormal signal intensity in the initial imaging (arrow). (C) Sagittal diffusion-weighted image and (D) ADC map show diffusion restriction in the anterior cord from C3 to C5 (arrows).

Fourteen days after the onset of symptoms, we performed a fresh MRI of the spine (Figure 2). The signal abnormality had extended from the cervicomedullary junction to T3. The diffusion-weighted images showed diffusion restriction in the anterior cord from C3-C5 at the site of the primary signal abnormality that was present in the initial MRI, with enhancement in the same location but not in the rest of the cord.

On discharge, on the 15th day of admission, the patient was clinically stable, though his neurological improvement was still slight. After three months of home physiotherapy, he showed significant improvement in strength, more in the right upper and lower limbs than in the left. He was now able to use his right hand for some self-care activities. Control of urine was still partial.

The patient’s parent provided informed consent for the publication of this case.

## Discussion

Fibrocartilaginous embolism is rarely reported as a cause of spinal cord infarction. Its acute presentation and manifestation render it hard to differentiate it from other causes, such as transverse myelitis and demyelinating disease, which, in turn, can lead to suboptimal management [3].

Our literature search for the keyword "fibrocartilaginous embolism" on the PubMed database returned 39 case reports featuring 49 FCE patients, half (44.9%) of whom were below the age of 20. More than half the patients (55%) were female.

All the 49 cases featured acute presentations. Our patient’s symptoms also began with a sudden onset of mild neck pain followed by a progressive paralysis. Several cases reported progression to respiratory insufficiency requiring high dependency or even intensive care, especially where the upper spine was involved, as in ours [4,5]. Four kinds of triggers were identified in these cases: sports (37.5%), heavy lifting (14.6%), minor trauma (8.3%), spinal surgery (2.1%), and idiopathic triggers (37.5%).

Similar to our case, almost all the reviewed cases reported flaccid paralysis and hypotonia. The majority of those patients presented with sensory manifestations, as did our patient [6,7,8]. Most FCE patients described in the literature had bowel or bladder incontinence or both, as did our patient [3,4,8,9].

Being rarely encountered in clinical practice, FCE clinical presentations being mistaken for other differential diagnoses such as transverse myelitis, Guillain-Barre syndrome, multiple sclerosis, and other neurological diseases [6,10]. We faced this dilemma in the current case, and what eventually led to the correct diagnosis was the poor response to immunotherapy and the late revelation from the patient that he had lifted heavy objects on the day of the incident. After carefully re-reviewing the MRI, fibrocartilaginous embolism leading to spinal cord infarction was considered the most likely cause. Of all the current investigation modalities, MRI is the most helpful in diagnosing FCE in live patients. In post-mortem investigations, however, autopsy examinations provide the most sensitive and accurate diagnosis [11].

The typical MRI findings of a cord infarct are cord swelling with increased T2 signal intensity [12]. The distribution of the signal abnormality is demonstrated in the axial T2 images and depends on the vascular territory involved [13]. The blood supply to the spinal cord is provided primarily by a single anterior spinal artery and paired posterior spinal arteries. The anterior spinal artery supplies the anterior two-thirds of the spinal cord, whereas the posterior spinal arteries supply the posterior third [14]. Owl’s eye appearance is a classic neuroimaging pattern seen in patients with anterior spinal artery infarct, representing T2 hyperintensity in the anterior horns in the axial images [15]. In our patient, the signal abnormality was distributed in the central gray matter with the involvement of the anterior and posterior horns. Early MR imaging with routine T2-weighted sequences may reveal no abnormality, and therefore, diffusion-weighted images should be included when cord ischemia is suspected [16]. The previous reports show that the presence of Schmorl nodes or disc collapse is highly suggestive of FCE [12]. In our patient, there was disc desiccation with a posterior annular fissure at the C2-3 level. The constellation of the clinical and imaging features is eventually diagnostic of FCE and makes other differentials of myelitis less likely. Comparison of the initial and follow-up images can show the evolution of the findings with the enhancement of the cord in the subacute phase due to the breakdown of the blood-cord barrier [8]. The fourteen-day follow-up MRI of our patient showed such changes, strengthening the diagnosis of FCE.

In many previous cases, the uncommon presentation of this condition led to initial misdiagnosis as transverse myelitis. Therefore, the initial management was often directed toward plasma exchange and immunotherapy along with rehabilitation, with little or no benefit. Upon diagnosis of FCE, these patients were recommended physiotherapy, which led to significant improvements [4,9,17,18]. In the current case also, our initial treatment with plasma exchange and immunotherapy with little benefit. Upon the diagnosis of FCE, our recommendation of long-term rehabilitation with home physiotherapy led to significant improvement, albeit with noticeable deficits.

## Conclusions

Fibrocartilaginous embolism is a rare cause of acute spinal cord infarction. To avoid misdiagnosis and inappropriate treatment, the clinician should be aware of the possibility of FCE during the clinical presentation. During history taking, it is vital to seek information on any precipitating activities or events, however minor. Thereafter, careful neurological examination with emphasis on the sensory system is required. A major clue for anterior spinal artery infarction can be signs of involvement of ascending spinothalamic tract and sparing dorsal column tract function, with associated limb weakness. It is vital to review the MRI and to look for findings suggestive of FCE-like desiccation of the intervertebral disc with annular fissure, as well as any abnormality in the intra-medullary signal, mainly in the gray matter of the cord with relative sparing of the lateral and posterior columns.
